# Prediction of the Mechanism of Sodium Butyrate against Radiation-Induced Lung Injury in Non-Small Cell Lung Cancer Based on Network Pharmacology and Molecular Dynamic Simulations

**DOI:** 10.3389/fonc.2022.809772

**Published:** 2022-06-28

**Authors:** Xiao-zhen Zhang, Mao-jian Chen, Ping-ming Fan, Ting-shi Su, Shi-xiong Liang, Wei Jiang

**Affiliations:** ^1^ Department of Radiation Oncology, Guangxi Medical University Cancer Hospital, Nanning, China; ^2^ Department of Respiratory Oncology, Guangxi Medical University Cancer Hospital, Nanning, China; ^3^ Department of Medical Oncology, Sun Yat-sen University Cancer Center, State Key Laboratory of Oncology in South China, Collaborative Innovation Center for Cancer Medicine, Guangzhou, China; ^4^ Department of Breast-Thoracic Tumor Surgery, The First Affiliated Hospital of Hainan Medical University, Haikou, China

**Keywords:** radiation-induced lung injury, non-small cell lung cancer, network pharmacology, molecular docking, molecular dynamics simulation, sodium butyrate, target gene, signaling pathway

## Abstract

**Background:**

Radiation-induced lung injury (RILI) is a severe side effect of radiotherapy for non-small cell lung cancer (NSCLC) ,and one of the major hindrances to improve the efficacy of radiotherapy. Previous studies have confirmed that sodium butyrate (NaB) has potential of anti-radiation toxicity. However, the mechanism of the protective effect of NaB against RILI has not yet been clarified. This study aimed to explore the underlying protective mechanisms of NaB against RILI in NSCLC through network pharmacology, molecular docking, molecular dynamic simulations and *in vivo* experiments.

**Methods:**

The predictive target genes of NaB were obtained from the PharmMapper database and the literature review. The involved genes of RILI and NSCLC were predicted using OMIM and GeneCards database. The intersectional genes of drug and disease were identified using the Venny tool and uploaded to the Cytoscape software to identify 5 core target genes of NaB associated with RILI. The correlations between the 5 core target genes and EGFR, PD-L1, immune infiltrates, chemokines and chemokine receptors were analyzed using TIMER 2.0, TIMER and TISIDB databases. We constructed the mechanism maps of the 3 key signaling pathways using the KEGG database based on the results of GO and KEGG analyses from Metascape database. The 5 core target genes and drug were docked using the AutoDock Vina tool and visualized using PyMOL software. GROMACS software was used to perform 100 ns molecular dynamics simulation. Irradiation-induced lung injury model in mice were established to assess the therapeutic effects of NaB.

**Results:**

A total of 51 intersectional genes involved in NaB against RILI in NSCLC were identified. The 5 core target genes were AKT1, TP53, NOTCH1, SIRT1, and PTEN. The expressions of the 5 core target genes were significantly associated with EGFR, PD-L1, immune infiltrates, chemokines and chemokine receptors, respectively. The results from GO analysis of the 51 intersectional genes revealed that the biological processes were focused on the regulation of smooth muscle cell proliferation, oxidative stress and cell death, while the three key KEGG pathways were enriched in PI3K-Akt signal pathway, p53 signal pathway, and FOXO signal pathway. The docking of NaB with the 5 core target genes showed affinity and stability, especially AKT1. *In vivo* experiments showed that NaB treatment significantly protected mice from RILI, with reduced lung histological damage. In addition, NaB treatment significantly inhibited the PI3K/Akt signaling pathway.

**Conclusions:**

NaB may protect patients from RILI in NSCLC through multiple target genes including AKT1, TP53, NOTCH1, SIRT1 and PTEN, with multiple signaling pathways involving, including PI3K-Akt pathway, p53 pathway, and FOXO pathways. Our findings effectively provide a feasible theoretical basis to further elucidate the mechanism of NaB in the treatment of RILI.

## Introduction

Lung cancer is the most common cause of cancer-related death worldwide (1). Non-small cell lung cancer (NSCLC) is the most common subtype of lung cancer, accounting for about 85% (2). The vast majority of NSCLC are diagnosed at an advanced inoperable stage. Concurrent chemoradiotherapy (CHRT) is the standard treatment for locally advanced inoperable NSCLC. CHRT significantly improve the overall survival of advanced NSCLC, with a 5-year survival rate of approximate 30% (3). However, radiation therapy for NSCLC is usually accompanied with the radiation-induced lung injury (RILI) (4). RILI may cause severe dyspnea and chronic pulmonary fibrosis, resulting in poor quality of life and even death (5). Previous studies have reported that inflammatory factors, including transforming growth factor beta (TGF-β) and tumour necrosis factor alpha (TNF-α), and immunological cells such as T helper cells and macrophage played vital roles in the occurrence and progression of RILI (6, 7). However, the exact mechanism of RILI is still unclear. Currently, the main treatment strategy for RILI is the combination of glucocorticoids and antibiotics, but the efficacy is limited. Additionally, the treatment of RILI require a long-term use of glucocorticoids, which may raise severe side effects (7). Therefore, there is an urgent need to explore the underlying mechanism of RILI, and develop effective drugs for RILI treatment.

Sodium butyrate (NaB) is a kind of short-chain fatty acid generated from the fermentation of dietary fibers by anaerobic bacterial within the colon (8). In addition, NaB is confirmed as a histone deacetylase inhibitor (HDACi). Many studies have proved that some traditional Chinese medicines can protect against RILI (9, 10), and NaB has also been reported to reduce radiation toxicity. Lee et al. (11) has demonstrated that NaB could alleviate radiation-induced cognitive dysfunction. Previous studies have shown that NaB could improve the efficacy of radiotherapy without damaging normal mucosa (12). Perona et al. (13) has reported that intraperitoneally administration of NaB would optimize the irradiation results. It is worth mentioning that inflammation is the most vital feature of acute lung injury (AIL) and RILI. A large number of previous studies have confirmed that NaB has extensive anti-inflammatory and immunomodulatory effects (14–16). NaB was shown to markedly downregulate the levels of interleukin 1β (IL1β) and TNF-α, and suppress the expression of nuclear factor κB, to attenuate immune response and relieve severe disruption of lung tissue structural (17). Additionally, the anti-tumor effect of NaB was revealed (18–20). In our previous study, we found that the combined therapy of NaB and docetaxel can additively inhibit proliferation and promote apoptosis of lung adenocarcinoma cells (21). Although accumulating evidences mentioned above indicated that NaB has both anti-radiological toxicity and anti-inflammatory effects for RILI, the mechanism by which NaB protected NSCLC from RILI has not been clarified.

Network pharmacology is a new discipline based on the theory of systems biology and multi-direction pharmacology, which can predict the pharmacological mechanism of drugs in disease through identifying multiple potential targets and signaling pathways (22, 23). Molecular docking and molecular dynamics simulation (MDs) are mainly used to predict the binding capability and stability of drug and target genes, and realize the virtual screening of the binding complex with drug and target gene (24–26). In this study, we employ network pharmacology, molecular docking, MDs, and *in vivo* experiments to explore the underlying mechanisms of NaB for the treatment of RILI in NSCLC.

## Methods

### Schematic Diagram of the Bioinformatic Analysis

**Figure 1:**
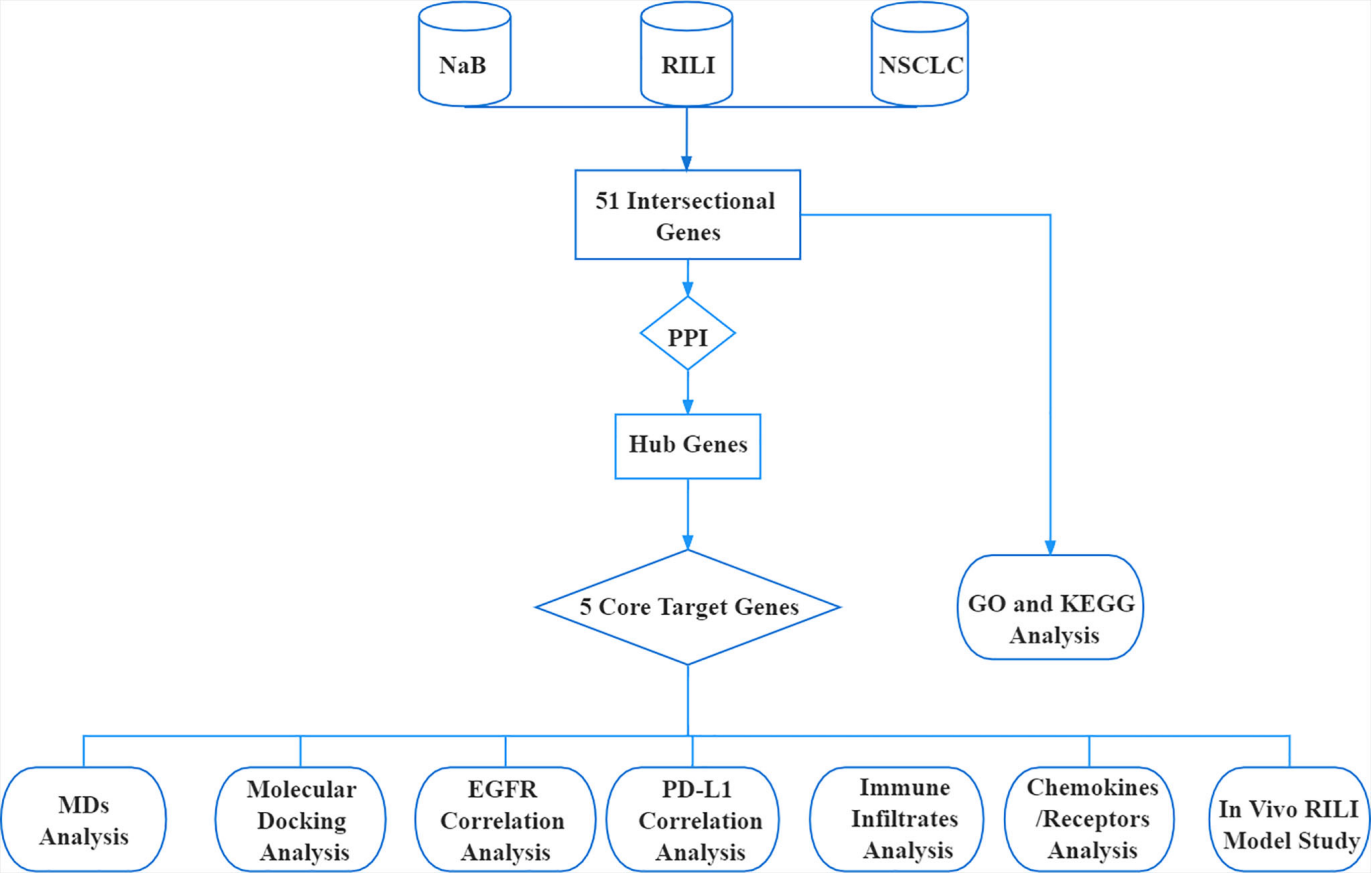
Flowchart of bioinformatic analysis.

The research procedure of our bioinformatic analysis is shown in the flowchart (**Figure 1**). First, we identified 51 intersectional genes associated with NaB, RILI and NSCLC. Second, based on the protein-protein interaction network (PPI) of the 51 intersectional genes, 10 hub genes were further screened out. Subsequently, 5 core target genes were identified as target genes of NaB against RILI. Also, the 51 intersectional genes were performed GO enrichment analysis and KEGG analysis. The relationships of the 5 core target genes with EGFR, PD-L1, immune cells infiltration, chemokines and chemokine receptors were investigated. In the end, the 5 core target genes were performed molecular docking and MDs analyses.

### Search and Identification of Common Targets

First, the 2D structural information of NaB (CAS:156–54-7) was downloaded from the NCBI PubChem (https://pubchem.ncbi.nlm.nih.gov/), and entered into the PharmMapper database (http://www.lilab-ecust.cn/pharmmapper/) to predict the potential targets. The names of target genes found from the PharmMapper database were converted to the formal gene names using the UniProt database (https://www.uniprot.org/). To optimize the identification of NaB target genes, we also conducted a literature retrieval, and excluded duplicates. The retrieval term was “(sodium butyrate [Title/Abstract]) AND (gene [Title/Abstract])”, and the retrieval time was limited to 2016-2021. The involved genes of RILI and NSCLC were identified from Online Mendelian Inheritance in Man (OMIM) (http://omim.org/) and GeneCards database (https://www.genecards.org/). Finally, we obtained 51 intersectional genes for NaB, NSCLC and RILI through the intersection of Venny 2.1 tool (http://bioinfogp.cnb.csic.es/tools/venny/index.html).

### Protein-Protein Interaction Network Construction and Hub Genes Screening

The 51 intersectional genes were uploaded to the STRING database (https://string-db.org/) to yield an interaction network. The protein type was chosen as “Homo sapiens” and the other parameters were set to default values. The protein interaction network files were imported into Cytoscape 3.8.2 software. The top 10 hub genes were screened by CytoHubber plug-in and MCC algorithm in Cytoscape 3.8.2 software. According to the values of “Degree”, “Betweenness Centrality” and “Closeness Centrality”, we identified the first 5 core target genes.

### Correlation Analyses Between the Top 5 Core Genes and EGFR, PD-L1, Immune Infiltrates, Chemokines and Chemokine Receptors

Based on the analysis of TIMER 2.0 (http://timer.cistrome.org/), TIMER (https://cistrome.shinyapps.io/) and TISIDB (http://cis.hku.hk/TISIDB/) databases, we further investigated the correlations between the 5 core target genes and EGFR, PD-L1, immune infiltrates, chemokines and chemokine receptors in lung adenocarcinoma (LUAD) and lung squamous cell carcinomas (LUSC) which are both the most common types of NSCLC.

### GO and KEGG Pathway Enrichment Analyses

The 51 intersectional genes were imported into the Metascape database (https://metascape.org/) to perform GO and KEGG enrichment analyses, and bubble maps were drawn *via* bioinformatics online tool (http://www.bioinformatics.com.cn/). According to the degree of genes enrichment and p-value, we screened out the top 12 most likely KEGG signaling pathways (*p*<0.0001) from the first 20, and identified target genes enriching in these pathways. The identified genes were uploaded to Cytoscape 3.8.2 software to construct a “component-target-pathway” map. Then, 3 key signaling pathways closely associated with RILI were screened out, and visualized using the KEGG database (https://www.genome.jp/kegg/).

### Molecular Docking Analysis

Subsequently, the best protein crystal structures of the 5 core target genes were downloaded from the RCSB PDB (https://www.rcsb.org/) database. These proteins were operated to remove water molecules, add polar hydrogen, and build active pockets using the AutoDock Vina tool (27). The 3D structure of NaB was imported into Chem3D 17.1 for optimization. In the AutoDock module, we ran to dock NaB to the proteins of 5 core target genes for 100 times using the Lamarckian Genetic Algorithm (LGA). Other parameters were set to the default values. The lowest binding energy for molecular docking was taken as the final result and visualized by PyMOL software. AKT1 with the lowest docking scores were used for the subsequent MDs.

### Molecular Dynamic Simulations Analysis

To further verify the reliability of docking results, GROMACS software was used to perform molecular dynamics simulation (MDs) analysis of AKT1 and NaB compounds. Before proceeding with the simulation, the general AMBER force field (GAFF) was used for substrates, while the partial atomic charges were obtained from the RESP method by Multwfn (28, 29). The missing parameters for the ligands were generated by the parmchk utility from AMBER tools. Na^+^ ions were added into the protein surface to neutralize the total charges of the systems. The systems were solvated in a rectangular box of TIP3P waters extending up to minimum cutoff of 15 Å from the protein boundary. The steepest descent and conjugate gradient method were used to optimize the energy of the initial structure. Then under canonical ensemble for 0.05 ns, the system was gently annealed from 10 to 300 K with a weak restraint of 15 kcal/mol/Å. Under isothermal-isobaric ensemble at target pressure of 1.0 atm and target temperature of 300K, 1 ns of density equilibration was performed by Langevin-thermostat and Berendsen barostat with pressure-relaxation time of 0.001 ns and collision frequency of 0.002 ns. After minimizations and equilibrations, MDs run of 100 ns was performed for ATK1-NaB complex systems using GROMACS software (30). Finally, according to the analysis of the GROMACS software, we get the corresponding root mean square deviation (RMSD) and root mean square fluctuation (RMSF), which can be used to evaluate the stability of ATK1-NaB complex system.

### *In Vivo* RILI Model and Experimental Design

Female wild-type C57BL/6 mice (8 weeks; 20–22 g) were purchased from the Experimental Animal Center of Guangxi Medical University (Nanning, China) and raised under specific pathogen-free condition. All procedures involving animals were approved by the Guangxi Medical University Experimental Animal Committee, and were performed in accordance with local and International Animal Welfare Guidelines.

In conducting an experiment, mice were randomly divided into three groups: Group I (Control group) received saline intraperitoneal administration but without irradiation treatment; Group II (IR group) received radiotherapy combined with saline treatment at each time point as NaB. Group III (NaB+IR group) received an intraperitoneal administration of NaB (Sigma-Aldrich, Shanghai, China) half an hour before irradiation at a dose of 500 mg/kg/day dissolved in saline and consolidating for seven consecutive days. The dosage of NaB was referred to previous publication (31). The single irradiation dose in lung was 15 Gy and dose rate was 1 Gy/min using 60 Co γ-rays, referring to previous research (32). Before radiation treatment, mice were anesthetized by isoflurane inhalation and then shielded with lead bricks to protect their head, abdomen, and extremities from radiation. The mice were euthanized after seven days and their lung tissues excised and harvested for further study.

### Histological Examination

Lung tissues of sacrificed mice were fixed with formalin, embedded in paraffin, then sliced into 5 μm section. Subsequently, the sections were subjected to standard hematoxylin and eosin staining to assess the histopathologic changes in lung tissue under a light microscope.

### Western Blot

Lung tissues were homogenized in RIPA lysis buffer (Beyotime Biotechnology, Shanghai, China) containing protease and phosphatase inhibitor cocktail (Beyotime Biotechnology, Shanghai, China). BCA protein assay kit (Beyotime Biotechnology, Shanghai, China) was utilized to measure the protein concentration. An aliquot of protein was separated by SDS-PAGE, and transferred onto polyvinylidene difluoride (PVDF) membranes. Blocked with QuickBlock™ Western Blot Blocking Buffer (Beyotime Biotechnology, Shanghai, China) at room temperature for 30 min, the membranes were incubated with specific antibodies at 4°C overnight, including anti-p-PI3K (1:1000), anti-PI3K (1:1000), anti-p-AKT (1:2000), anti-AKT (1:1000) (All from Cell Signaling Technology, Danvers, MA, USA) and anti-GAPDH (1:10000) (Abcam, Cambridge, MA, USA). After washing 3 times with tris-buffered saline containing Tween 20 (TBST), the membranes were incubated with the corresponding HRP-conjugated secondary antibody (EarthOx Life Sciences, Millbrae, CA, USA) at room temperature for 1 h. After washing 3 times with TBST, the immunoreactive protein bands were determined by luminescent visualization using an enhanced chemiluminescence reagent ECL kit (Beyotime Biotechnology, Shanghai, China). The signal intensity was measured using enhanced chemiluminescence detection system (BioRad, Hercules, CA, USA).

## Results

### The Search and Identification of Common Genes for Sodium Butyrate, RILI, and NSCLC

**Figure 2:**
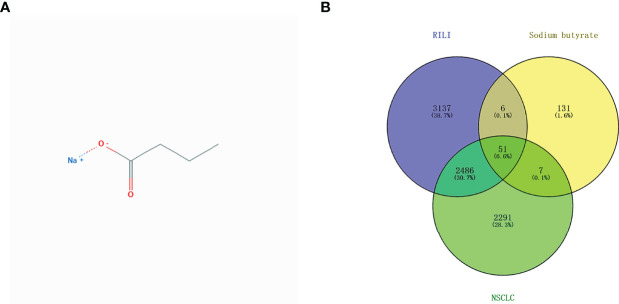
2D structural information of sodium butyrate **(A)**; Venn diagram of involved genes of sodium butyrate, RILI and NSCLC **(B)**.

The 2D structural information of NaB was obtained from the NCBI PubChem database (**Figure 2A**). A total of 196 NaB target genes were predicted, while 4839 genes involved in NSCLC, and 5681 genes in RILI were identified. A total of 51 intersectional genes are thought to be involved in the mechanism of NaB against RILI (**Figure 2B**). Then, the 51 intersectional genes were used for subsequent research.

### Protein-Protein Interaction Network and Hub Genes

**Figure 3:**
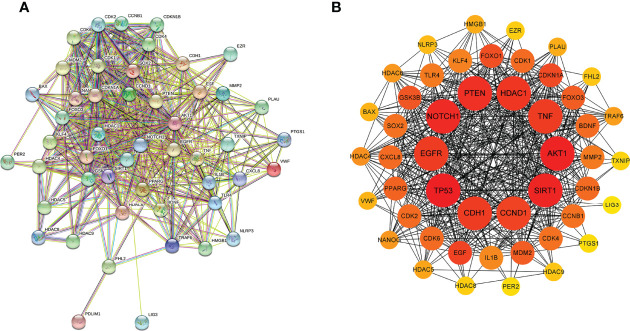
Protein-protein interaction (PPI) network of the 51 intersectional genes was analyzed by STRING database **(A)** and the hub targets network including the top 10 targets were constructed by cytoHubba plug-in **(B)**. In the hub targets network, the larger the node and the redder the color represent the stronger the interaction degree.

As shown in **Figure 3A**, the PPI network displayed the interaction of the 51 intersectional genes. Hub genes network diagram further showed the interaction degree of the 51 intersectional genes, and identified the top 10 hub genes, including AKT1, TP53, NOTCH1, SIRT1, PTEN, CCND1, CDH1, EGFR, HDAC1 and TNF (**Figure 3B**). The larger the node and the redder the color represent the stronger the interaction degree. Then, 5 core target genes were screened out based on the “Degree” algorithm, including AKT1, TP53, NOTCH1, SIRT1 and PTEN. These 5 core genes were considered to be the most likely target genes for the protective effect of NaB against RILI.

### Correlations Between the Top 5 Core Genes and EGFR, PD-L1, Immune Infiltrates, Chemokines and Chemokine Receptors

**Figure 4:**
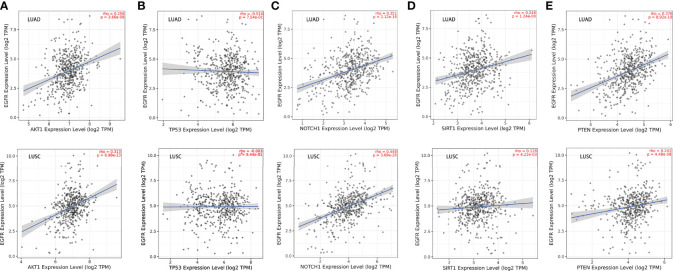
Correlations between EGFR and the 5 core target genes in LUAD and LUSC were studied using TIMER 2.0 database. **(A)** AKT1-EGFR. **(B)** TP53-EGFR. **(C)** NOTCH1-EGFR. **(D)** SIRT1-EGFR. **(E)** PTEN- EGFR.

**Figure 5:**
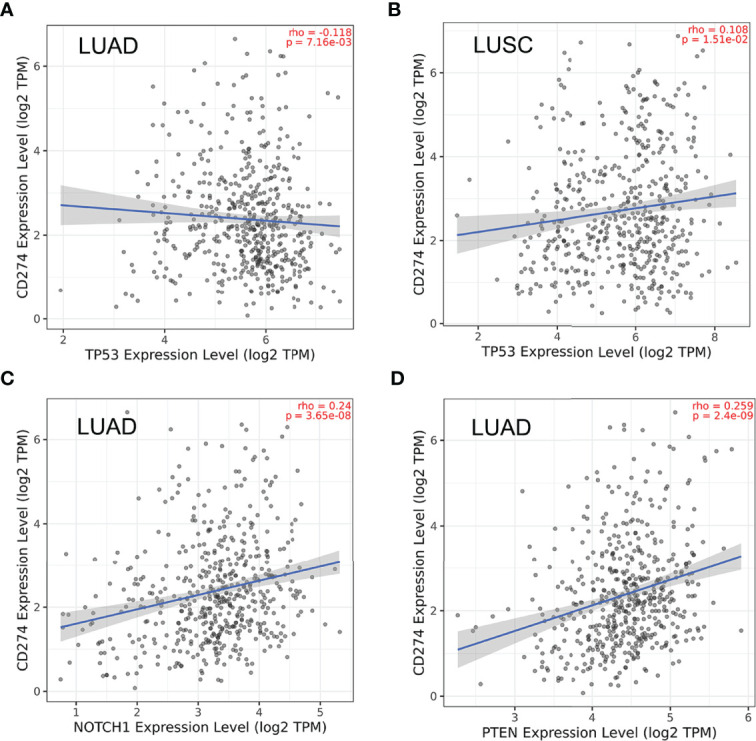
Correlations between PD-L1 (CD274) and the 5 core target genes were evaluated using TIMER 2.0 database. **(A)** TP53-PD-L1 in LUAD. **(B)** TP53-PD-L1 in LUSC. **(C)** NOTCH1-PD-L1 in LUAD. **(D)** PTEN-PD-L1 in LUAD.

Given that previous studies have reported that epidermal growth factor receptor-tyrosine kinase inhibitors (EGFR-TKIs) combined with radiotherapy were more inclined to develop RILI (33, 34), we further explored the correlation between the 5 core target genes and EGFR using the TIMER 2.0 database. We found that the expression of EGFR was closely associated with AKT1, NOTCH1, SIRT1 and PTEN (*P*<0.05), but not TP53 (*P*>0.05) (**Figure 4**). It has been confirmed that the use of PD-L1 inhibitors increase the risk of RILI (35). We further investigated the correlation between PD-L1 and the 5 core target genes. The result based on the TIMER2.0 database showed that the expression of PD-L1 was significantly associated with the expressions of TP53 (*P*<0.05) in LUAD and LUSC, NOTCH1 (*P*<0.05) in LUAD, and PTEN (*P*<0.05) in LUAD (**Figure 5**).

**Figure 6:**
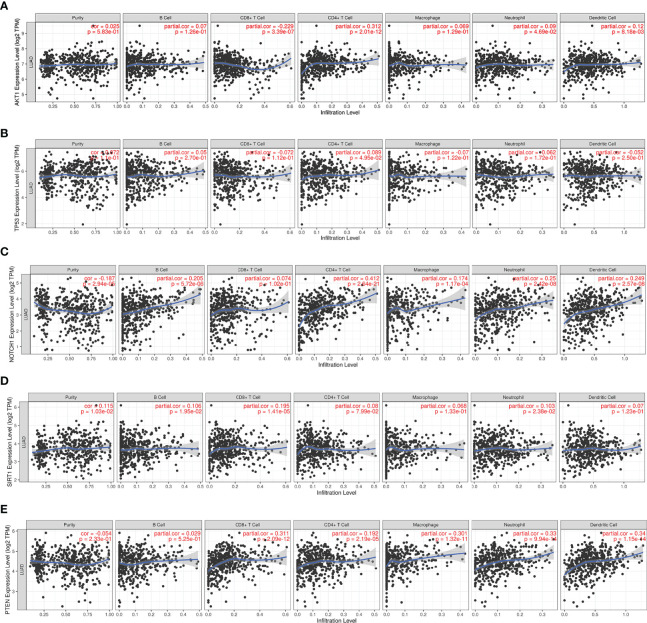
Correlations between the 5 core target genes and immune infiltration level in LUAD were analyzed using TIMER database. **(A)** AKT1. **(B)** TP53. **(C)** NOTCH1. **(D)** SIRT1. **(E)** PTEN.

**Figure 7:**
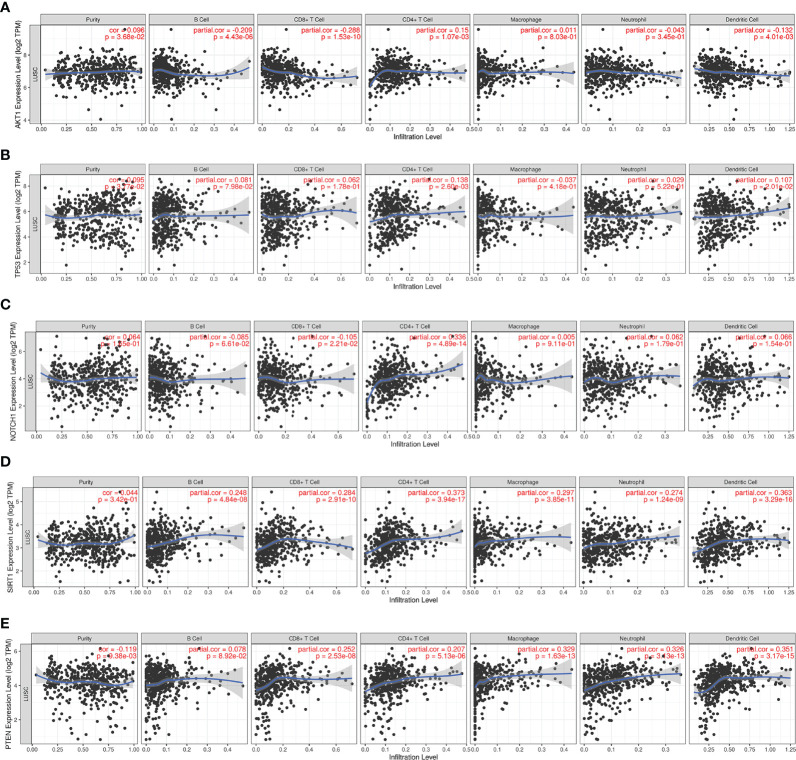
Correlations between the 5 core target genes and immune infiltration level in LUSC were analyzed using TIMER database. **(A)** AKT1, **(B)** TP53, **(C)** NOTCH1, **(D)** SIRT1, **(E)** PTEN.

**Figure 8:**
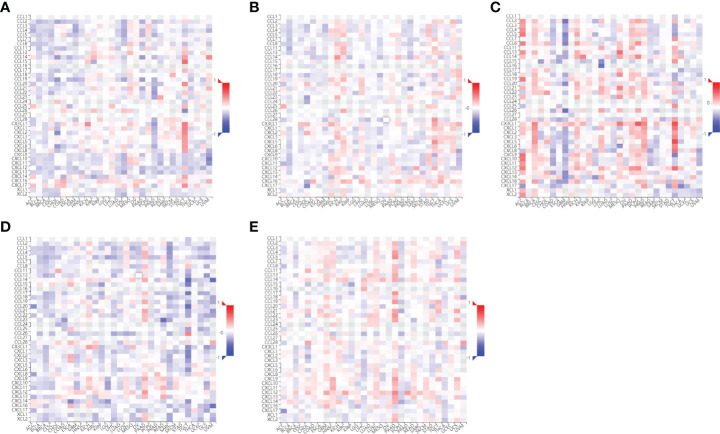
Correlations between chemokines and the 5 core target genes were explored using TISIDB database. **(A)** AKT1-chemokines. **(B)** TP53-chemokines. **(C)** NOTCH1-chemokines. **(D)** SIRT1-chemokines. **(E)** PTEN-chemokines.

**Figure 9:**
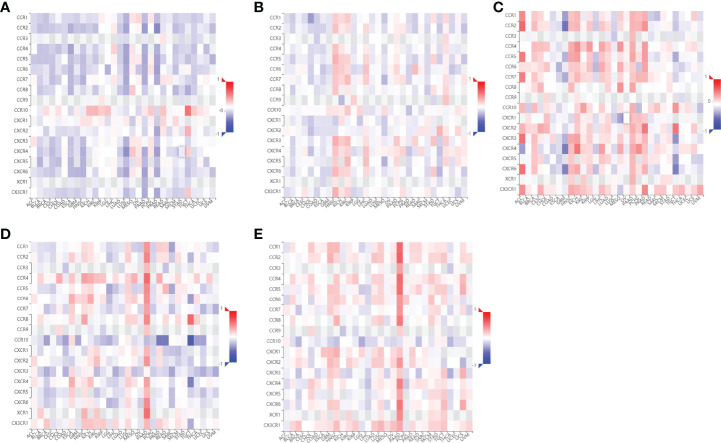
Correlations between chemokine receptors and the 5 core target genes were analyzed using TISIDB database. **(A)** AKT1-chemokines receptors. **(B)** TP53-chemokines receptors. **(C)** NOTCH1-chemokines receptors. **(D)** SIRT1- chemokines receptors. **(E)** PTEN-chemokines receptors.

Since RILI is closely correlated to immune and inflammatory reaction (6), further analyses using TIMER and TISIDB databases showed that the expressions of the 5 core target genes were associated with immune infiltrations in LUAD (**Figure 6**) and LUSC (**Figure 7**), to a certain extent. For example, the expression of NOTCH1 was closely associated with B cell, CD4^+^T cell, macrophage, neutrophil and dendritic cell in LUAD (P<0.05) (**Figure 6C**); PTEN was significantly associated with CD8^+^T cell, CD4^+^T cell, macrophage, neutrophil and dendritic cell in LUAD (P<0.05) (**Figure 6E**); and SIRT1 was associated with B cell, CD8^+^T cell, CD4^+^T cell, macrophage, neutrophil and dendritic cell in LUSC (P<0.05) (**Figure 7D**). In addition, the expressions of AKT1, TP53, NOTCH1 and SIRT1 were mainly negatively correlated with most chemokines and chemokine receptors in either LUAD or LUSC (**Figures 8**, **9**); while the expression of PTEN was mainly positively associated with chemokine receptors (**Figure 9E**).

### GO and KEGG Pathway Enrichment Analysis

**Figure 10:**
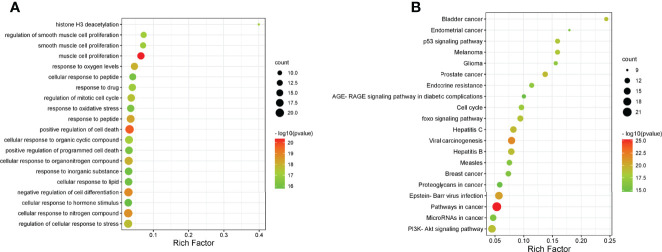
GO analysis and KEGG analysis of the 51 intersectional genes were performed using Metascape database. **(A)** GO analysis of target genes. **(B)** KEGG pathway analysis of target genes.

**Figure 11:**
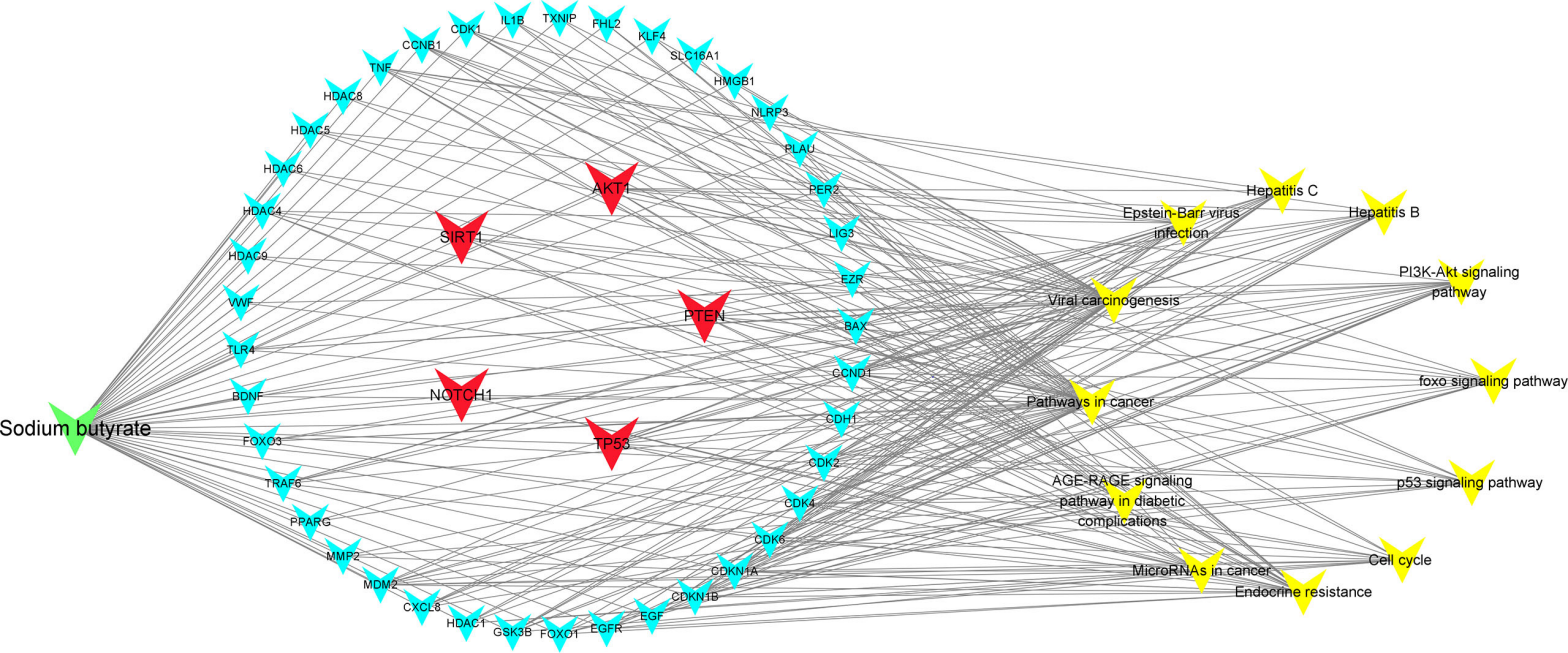
Compound-targets-pathways map was constructed using Cytoscape 3.8.2 software. The compound (sodium butyrate) was green node, targets were blue and red nodes, and pathways were showed by yellow nodes, respectively. The red represents the 5 core target genes. The edges represent the interactions among them.

**Figure 12:**
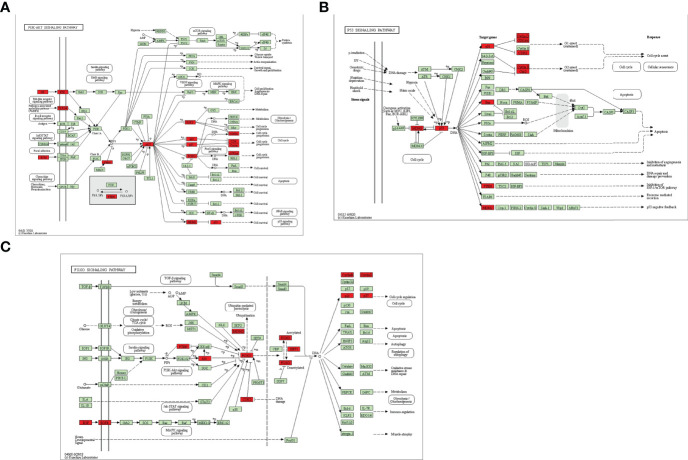
Key KEGG signaling pathways were visualized using the KEGG database. **(A)** PI3k-Akt signaling pathway. **(B)** p53 signaling pathway **(C)** FOXO signaling pathway. Red rectangles represent some of the target genes of the 51 intersectional genes. Green rectangles indicate unidentified proteins.

To further explore the underlying function and mechanism of the 51 intersectional genes, we performed GO and KEGG pathway enrichment analysis. As shown in **Figure 10A**, the results showed that the top 20 biological processes (BPs) were mainly focused on the regulation of smooth muscle cell proliferation, oxidative stress and cell death, etc. The top 20 KEGG pathways mainly included several pathways involved in cancer, the PI3K-Akt signaling pathway, p53 signaling pathway, FOXO signaling pathway, viral carcinogenesis, Epstein-Barr virus infection, cell cycle, microRNAs in cancer, etc (**Figure 10B**). We created a “component-target-pathway” map to exhibit the effect of NaB on the 51 intersectional genes and signaling pathways against RILI using the Cytoscape software (**Figure 11**). The most likely signaling pathways that NaB improved RILI were PI3K-Akt signaling pathway, p53 signaling pathway, and FOXO signaling pathway, based on genes enrichment degree and p-value. The p-values of all these 3 pathways are less than 0.0001. The maps of these 3 key pathways were utilized to explain the mechanism of NaB against RILI (**Figure 12**).

### Molecular Docking

**Figure 13:**
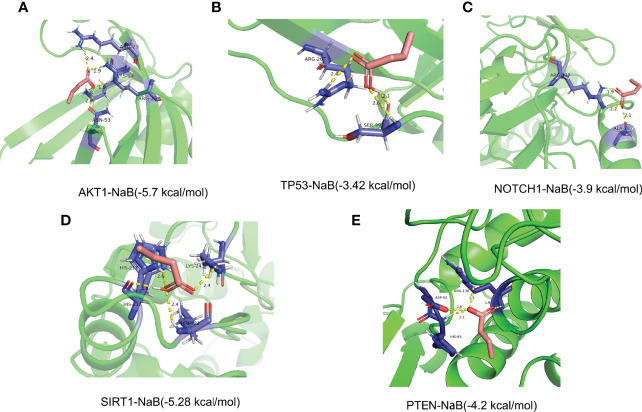
Stereogram and docking energy score of molecular docking using AutoDock Vina tool and PyMOL software. **(A)** AKT1-NaB. **(B)** TP53-NaB. **(C)** NOTCH1-NaB. **(D)** SIRT1-NaB. **(E)** PTEN-NaB. The yellow dotted line represents the interaction between NaB and the target protein. NaB, sodium butyrate.

To evaluate the binding ability of NaB to the 5 core target genes, we performed molecular docking and plotted images. Lower score of binding energy indicates stronger binding affinity of the docked complex; while binding energy < 0 kcal/mol indicates that ligand molecules can spontaneously bind to the receptor proteins (25, 27, 36). Our results revealed that the binding energies of NaB binding to the 5 core target genes were all less than 0 kcal/mol. Specifically, the scores of binding energies are -5.7 kcal/mol (AKT1-NaB), -3.42 kcal/mol (TP53-NaB), -3.9 kcal/mol (NOTCH1-NaB), -5.28 kcal/mol (SIRT1-NaB), and -4.2 kcal/mol (PTEN-NaB), respectively, indicating a high affinity between NaB and these 5 core target genes, in particular AKT1-NaB (**Figures 13A–E**).

### MDs

**Figure 14:**
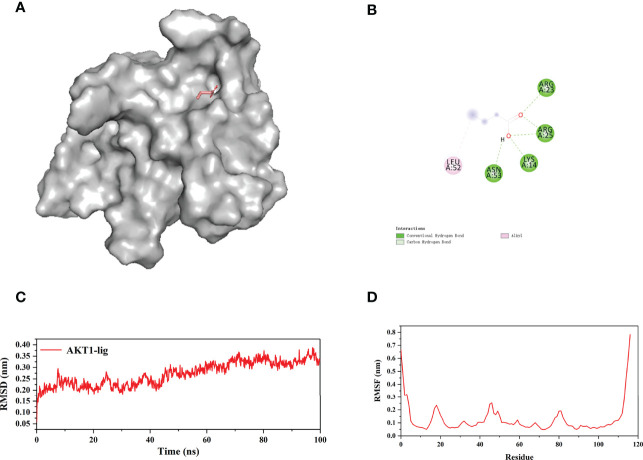
100 ns molecular dynamic simulations of AKT1-NaB using the GROMACS software. **(A)** The 3D stereoscopic molecular map of AKT1 and NaB docking. **(B)** 2D display of water and hydrogen bond docking between AKT1 and NaB. **(C)** RMSD and **(D)** the RMSF of molecular dynamic simulations. NaB, sodium butyrate.

Since the flexibility of the protein and the solvent environment are not considered in the molecular docking process (**Figures 14A, B**), we further verify the reliability of the docking results through molecular dynamics simulations (MDs). Considering that AKT1 has the lowest docking energy with NaB, AKT1 was selected for 100 ns MDs. The RMSD was used to judge whether the AKT1 complex system reaches equilibrium during the simulation process. Generally, a smaller RMSD value indicates a more stable system (37). As shown in **Figure 14C**, the RMSD curve fluctuated around 2.7Å and the amplitude remained within 3.5 Å, indicating that the AKT1-NaB complex system was stable and the bond was firm. We used RMSF to evaluate the stability of each amino acid of AKT1 protein in complex system. Except for the loop region at both ends of the AKT1 protein, the RMSF curve fluctuated within 2.5Å, confirming the strong stability of AKT1-NaB complex (**Figure 14D**). In brief, MDs further verified that AKT1-NaB compound was stable and tightly combined.

### NaB Treatment Alleviates Radiation-Induced Lung Injury

**Figure 15:**
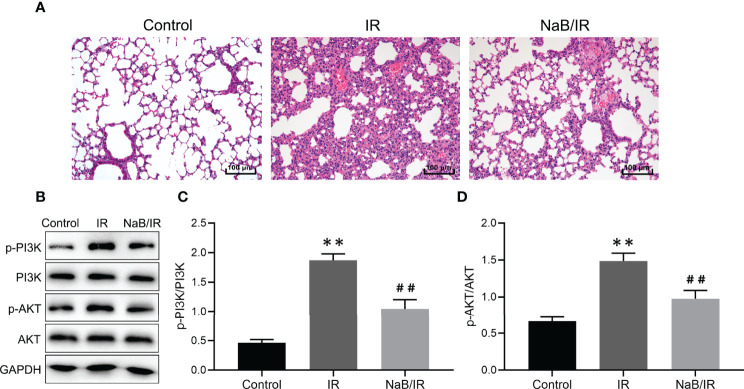
Sodium butyrate treatment attenuates radiation-induced lung injury. **(A)** Hematoxylin and eosin staining of lung tissues 1 week after radiation administration. Scale bar 100 μm. **(B)** The protein levels of p-PI3K, PI3K, p-AKT and AKT in lung tissue were detected by Western blot. **(C, D)** Quantitative analysis of **(B)**. Results are representative of three independent experiments, and the differences between data were evaluated by one-way ANOVA, with *p* value less than 0.05 considered statistical significance. **p < 0.001 *vs.* Control; ^##^ *p* < 0.05 *vs.* IR.

To determine the effect of NaB on RILI, the mice were intraperitoneal injected with 500 mg/kg NaB pre- and post-radiotherapy. After 1 week of a single dose of 15Gy local irradiation inducing lung local radiation model, we collected lung tissues for histological evaluation. As shown in **Figure 15A**, mice in control group showed no significantly destruction in lung tissues; while lung tissues from mice in IR group demonstrated obviously interstitial congestion and edema, with thickened alveolar walls and collapsed alveolar. With NaB (500 mg/kg) treatment, these pathological changes were significantly reversed. Taken together, these results suggested that NaB treatment alleviates RILI.

### NaB Treatment Inhibits PI3K/AKT Signaling Pathway in Radiation-Induced Lung Injury

Our bioinformatic results reveal that PI3K/AKT pathway maybe a key signaling pathway involved in NaB against RILI. Then, we further investigated the modulatory effect of NaB on PI3K/AKT pathway. As seen in **Figures 15B–D**, the phosphorylation levels of PI3K and AKT in IR group were increased compared to control group. NaB treatment significantly reversed the radiation-induced phosphorylation of PI3K and AKT. Our results revealed that NaB treatment may protect against RILI by inhibiting the activation of PI3K/AKT signaling pathway.

## Discussion

Radiotherapy for thoracic malignancies is a standard therapeutic strategy in advance NSCLC (38). However, RILI is a severe side effect of radiotherapy with great impact on the curative effect of NSCLC, since it limits the radiation dose, a crucial factor involving in effective tumor killing (39, 40). Although growing evidence suggests that inflammation (41, 42), immune regulation (43, 44), and reactive oxygen species are involved in the occurrence and development of RILI (39), the exact mechanism is still unclarified, and there is no specific drug for treatment. It is extremely necessary to investigate the mechanism of RILI, and develop new drugs. NaB has been reported, by a large number of studies, that exerts potential activities of anti-radiation toxicity (11, 12), anti-inflammation (45–47), immunomodulation (48, 49), and anti-tumor (50–52). The anti-lung cancer effect of NaB has been proved in our preliminary study (21). However, the mechanism of the protective effect of NaB against RILI needs further exploration. In this study, among the 51 intersectional genes of NaB, RILI and NSCLC, 5 core target genes were identified and thought to be involved in the mechanism of NaB against RILI, including AKT1, TP53, NOTCH1, SIRT1 and PTEN. We revealed the close relationship between the 5 core target genes and immune cell infiltration, and most of the chemokines and chemokine receptors. GO analysis of the 51 intersectional genes exhibited that BPs were focused on the regulation of smooth muscle cell proliferation, oxidative stress and cell death. Whereas the three key KEGG pathways were enriched in the PI3K-Akt pathway, p53 pathway, and FOXO pathway. We further constructed a compound-target-pathway network, depicting the relationship between NaB and the involved genes and pathway of RILI. Finally, we demonstrated that the 5 core target genes have good affinity and stability with NaB. Our experimental *in vivo* also verified that NaB can improve RILI. Collectively, all the findings above showed that NaB may act on multi-target genes, multi-pathway, and multi-function to protect against RILI.

Through the PPI network and hub genes analyses of the 51 intersectional genes, AKT1, TP53, NOTCH1, SIRT1 and PTEN were identified as the most likely potential targets of NaB treatment for RILI. All these target genes have been confirmed to correlate to radiation toxicity. A study reported that phosphorylation of AKT increased in the lung of irradiated mice, and myrtol inhibited the phosphorylation of AKT to protect against RILI (53). The AKT-mediated pathway was significantly associated with RILI grade 3 (54). TP53 is a tumor suppressor gene associated with RILI. A study of Yang et al. (55) has shown that the polymorphisms of TP53 and ATM were associated with the risk of RILI in lung cancer patients treated with radiotherapy. Mathew et al. (10) reported that simvastatin could reverse RILI-associated dysregulated gene expression including TP53. Genetic alterations in NOTCH1 were associated with a high mean grade of radiation-induced toxicity in head and neck squamous cell carcinoma (56). SIRT1 has a protective effect against radiation injury (57). Additionally, Zhang et al. (58) reported that active PTEN signaling after radiation is closely related to RILI. Our result *in vivo* also confirmed the increased phosphorylation levels of AKT with radiotherapy, and NaB protect against radiation-induced lung injury with decreased phosphorylation of AKT. Collectively, all these genes, including AKT1, TP53, NOTCH1, SIRT1 and PTEN, may play a vital role in radiotoxicity, and we speculated that NaB treatment against RILI is associated with the regulation of these target genes.

Numerous studies have found that RILI is closely related to inflammatory cytokine and immune cells (6, 7). In our findings, the expressions of the 5 core target genes showed some relationship with either immune infiltrations or chemokines and chemokine receptors. Based on these findings, we further performed GO function and KEGG pathway analysis of target genes. The results from GO analysis revealed that the major biological processes were enriched in the regulation of smooth muscle cell proliferation, oxidative stress, and cell death. The identified 5 core target genes have also been reported to involve in these biological processes. Previous research has reported that AKT1 played an important role in the proliferation and migration of vascular smooth muscle cells (VSMCs) and anti-oxidative stress-induced apoptosis (59). Wu et al. (60) demonstrated that the reduction of ribosome biogenesis in aortic smooth muscle cells may lead to TP53-dependent proliferation inhibition, oxidative stress, and apoptosis. Xuan et al. (61) proved that NOTCH1-activated extracellular vesicles of cardiac mesenchymal stem cells promoted cardiomyocyte proliferation and angiogenesis. Liu et al. (62) found that SIRT1 could promote the proliferation of SK-N-SH cells, and protect them from cell death induced by oxidative stress. Sedding and colleagues founded the up-regulation of PTEN induced by oxidative stress in damaged vascular VSMCs (63). The biological processes related to the 5 core target genes further support the results of our GO analysis. Noteworthy, these biological processes are also closely related to the occurrence and development of RILI (64, 65). For example, hydrogen therapy was confirmed to attenuate irradiation-induced lung damage by reducing oxidative stress (64). Isoflavone have showed radioprotective effects in irradiated lungs by limiting excessive immune cell homing *via* vascular endothelium into damaged lung tissue (65). Taken together, NaB may act on these target genes to modulate biological processes including smooth muscle cell proliferation, oxidative stress, and cell death, thereby alleviating RILI.

The results from KEGG analysis indicated that multiple target genes of NaB were mainly enriched in the PI3K-Akt pathway, p53 pathway, and FOXO pathway, which were shown to play an essential role in radiotherapy. Our RILI model also showed significantly increased phosphorylation of PI3K and AKT with radiotherapy. Notably, NaB treatment suppress the activities of PI3K and AKT, and protect against RILI. Research has shown that severe RILI in lung cancer patients was associated with genetic variants in the PI3K-Akt signaling pathway (54). Repeated radon exposure induced lung injury by activating the PI3K/AKT/mTOR pathway (66). RILI can lead to chronic pulmonary fibrosis, which can be reduced by inhibition of the PI3K/AKT/mTOR pathway (67). Alleviation of radiation-induced DNA damage were associated with downregulating p53 mediated signaling pathway (68). Increased MMP-2 expression mediated by p53 is involved in RILI (69). Additionally, research showed that the baicalein inhibited radiation-induced inflammatory response through up-regulating FOXO activation, and down-regulating NF-κB (70). Moskalev et al. (71) demonstrated an essential role of FOXO in hormesis and radiation adaptive response which had a protective effect on the body. Overall, NaB may alleviate RILI by modulating the pathways mentioned above.

The limitations of this study include the following aspects. First, our results of involved genes and signaling pathways of RILI need a really world cohort to verify. Second, we still need further biological experiments to verify the pharmacological mechanisms of sodium butyrate.

## Conclusion

In conclusion, NaB may alleviate RILI through multiple target genes including AKT1, TP53, NOTCH1, SIRT1 and PTEN. In addition, multiple signaling pathways involved in the protective effect of NaB against RILI, including PI3K-Akt pathway, p53 pathway, and FOXO pathways. Hence, the mechanism of NaB against RILI is multi-target and multi-pathway. Our findings effectively provide a feasible theoretical basis for further elucidation of NaB in the treatment of RILI.

## Data Availability Statement

The original contributions presented in the study are included in the article/supplementary material. Further inquiries can be directed to the corresponding authors.

## Author Contributions

XZ: Methodology, Writing - original draft, Writing - review and editing. MC: Writing - review and editing. PF: Search information, review. TS: review and editing. SL: Writing - review and editing. WJ: Project administration, Funding acquisition, Resources, Writing - review and editing. All authors contributed to the article and approved the submitted version.

## Funding

The work was funded by Beijing Xisike Clinical Oncology Research Foundation (Y-2019AZQN-04532), and the Key Program of Science and Technology of Guangxi, China (AB20159024).

## Conflict of Interest

The authors declare that the research was conducted in the absence of any commercial or financial relationships that could be construed as a potential conflict of interest.

## Publisher’s Note

All claims expressed in this article are solely those of the authors and do not necessarily represent those of their affiliated organizations, or those of the publisher, the editors and the reviewers. Any product that may be evaluated in this article, or claim that may be made by its manufacturer, is not guaranteed or endorsed by the publisher.
